# Engineering the TCA cycle regulator GarA to increase erythromycin production in Saccharopolyspora erythraea

**DOI:** 10.1099/mic.0.001583

**Published:** 2025-08-04

**Authors:** Anna D. Liuzzi, Hannah L. Tompkins, Sarah K. Pallett, Lee Webster, Galina V. Mukamolova, Matthew A. Gregory, Martin Sim, Helen M. O'Hare

**Affiliations:** 1 Department of Respiratory Sciences, University of Leicester, University Road, Leicester LE1 7RH, UK; 2 Isomerase Therapeutics, Newnham Building, Chesterford Research Park, Little Chesterford, Saffron Walden, Cambridge, Cambridgeshire, CB10 1XL, UK

**Keywords:** actinobacteria, central metabolism, erythromycin, polyketide biosynthesis, protein kinase, TCA cycle

## Abstract

Actinobacteria are important for industrial production of antibiotics, fine chemicals and food and a source of new compounds for drug discovery. Their central metabolism is regulated by a conserved protein GarA that is unique to the Actinobacteria and has been studied in *Mycobacterium tuberculosis* and *Corynebacterium glutamicum*. GarA regulates the TCA cycle and glutamate metabolism by direct binding to enzymes to modulate their activity on glutamate and alpha-ketoglutarate. Given the importance of the TCA cycle in the synthesis of acyl-CoA precursors for antibiotic biosynthesis, and increasing evidence for the role of nitrogen regulators in control of secondary metabolism, we hypothesized that engineering GarA could be used to enhance production of valuable metabolites. His_6_-tagged GarA was introduced into *Saccharopolyspora erythraea*, an overproducer of the polyketide antibiotic erythromycin. Phosphorylation of GarA was detected at the N-terminal ETTS motif, suggesting that it is regulated by protein kinases like in *M. tuberculosis*. GarA expression was observed at all growth stages, and a truncated form lacking the phosphorylation site accumulated during late fermentation. Engineered *S. erythraea* expressing phosphoablative GarA produced twofold more erythromycin, both in standard fermentation broth and in minimal medium. To investigate the mechanism for the increased titre, the engineered strain was characterized for transcription of erythromycin biosynthetic genes, as well as its ability to metabolize glutamate and its intracellular and extracellular aa content. The observed alterations in aa metabolism are consistent with the role of GarA as a TCA cycle regulator that may influence precursor supply for polyketide biosynthesis.

## Introduction

The Actinomycetes, mainly *Streptomyces*, are the most important group of bacteria for producing bioactive compounds like antibiotics [1].

Drugs produced by Actinobacteria include antibiotics, anti-parasitic and anti-cancer and are categorized by their biosynthesis and chemical structure, for example, polyketides, aminoglycosides and non-ribosomal peptides.

An important bottleneck for drug discovery, development and commercialization is optimizing culture to achieve adequate yields [2]. To date, this has been achieved by optimization of fermentation conditions and feedstock, alongside strain improvement. Approaches to strain improvement by rational engineering have been investigated, such as upregulating expression of biosynthetic gene clusters, deletion of competing metabolic pathways and metabolic engineering. For example, increasing the acyl-CoA building blocks can enhance the production of polyketide antibiotics and has been achieved by adding propanol to the fermentation feedstock and by engineering genes for acyl-CoA metabolism [3,5].

We hypothesized that the conserved Actinobacterial metabolic regulator GarA could provide a route to improve the production of antibiotics as it influences the TCA cycle and nitrogen metabolism. GarA is unique to the Actinobacteria and appears to have co-evolved with a domain rearrangement of the oxoglutarate dehydrogenase (ODH) complex [6,8]. The ODH complex is subject to multiple levels of regulation to control the production of ATP and NADH, and GarA is itself controlled by conserved protein kinases PknB and PknG that respond to cell wall and nutritional stimuli [9,11]. GarA binds to the ODH complex to inhibit the TCA cycle, and it also inhibits glutamate entry to the TCA cycle and promotes glutamate synthesis [12]. Modification of GarA has a major impact on metabolism: deletion of *garA* reduced the ability to synthesize glutamate of *Mycobacterium tuberculosis*, *Mycobacterium smegmatis* and *Corynebacterium glutamicum* [13,14], whereas disruption of GarA phosphorylation (by deletion of the kinase, or engineering the phosphorylation sites of GarA) reduced the ability of these bacteria to catabolize aa and increased glutamate production [9,10, 13]. Rational mutation of protein kinases and *garA* in *C. glutamicum* has been applied to increase the fermentation yield of other valuable products as well as glutamate: arginine, GABA and 4-hydroxyleucine [15,17].

**Fig. 1.**
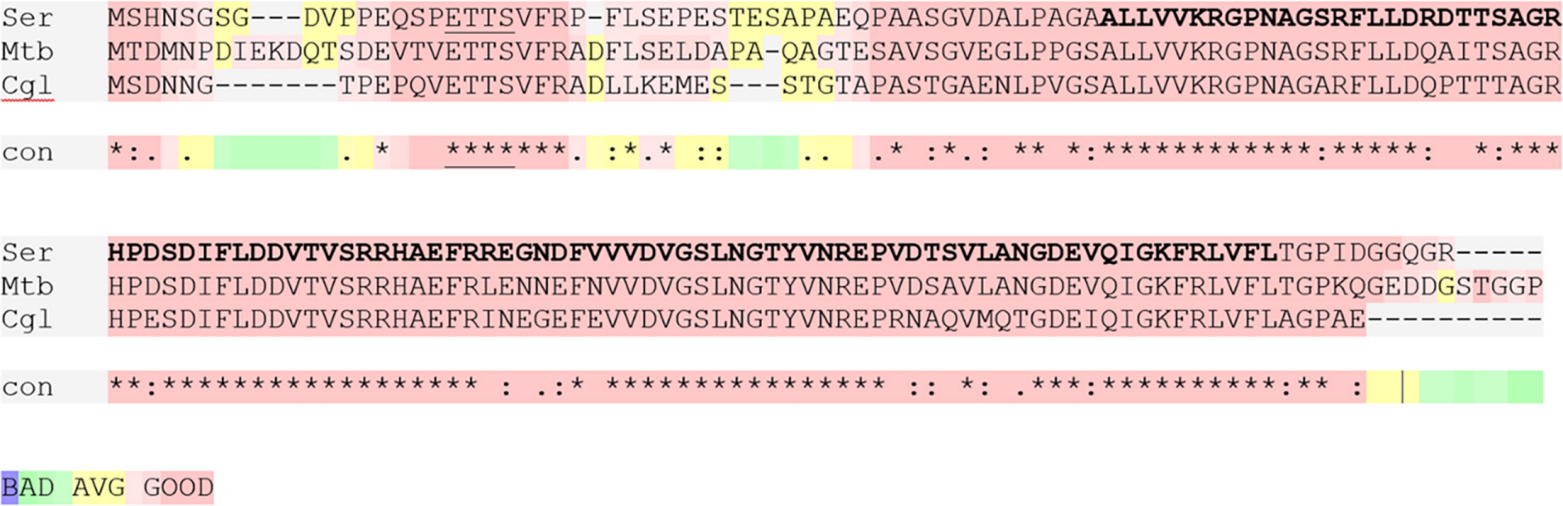
*Saccharopolyspora erythraea* GarA is encoded by SACE_3845 and shares high identity with characterized homologues. SACE_3845 was aligned with GarA from *M. tuberculosis* and *C. glutamicum*. The N-terminal ETTS phosphorylation motif is underlined, and the conserved FHA domain is in bold. GarA from *S. erythraea* shares 79% and 65% aa identity with these homologues, respectively, and 90% and 79% within the FHA domain.

GarA has been characterized in *Mycobacteriaceae* and *Corynebacteriaceae* for its effects on bacterial metabolism [6,10], structure and interaction with ODH [18,22] and regulation by protein kinases [6,7, 10, 11]. It is likely to have a conserved function in other Actinobacteria, including antibiotic producers, since it is highly conserved, particularly its binding site for ODH [on a forkhead-associated (FHA) phosphothreonine recognition domain] and its regulatory phosphorylation sites (two threonines in a conserved four aa motif, ETTS and N-terminal to the FHA domain) (Fig. 1).

An erythromycin-overproducer strain of *Saccharopolyspora erythraea* was chosen to investigate the potential of GarA for metabolic engineering to improve polyketide antibiotic yield. We report engineering of *S. erythraea* to increase GarA expression (encoded by SACE_3845) and introduce phosphoablative GarA (lacking the phosphorylation motif), the latter of which gave a twofold enhancement in erythromycin yield and showed phenotypes consistent with the inhibition of ODH activity and glutamate catabolism.

## Methods

### Bacterial strains and culture

*S. erythraea* used in this study is an overproducer derivative of NRRL2338 [23], termed ISOM-1717, and was cultured at 30 °C generally and 28 °C for fermentation. AS-1 broth agar was used for routine culture [1 g l^−1^ yeast extract (Fisher Scientific), 0.2 g l^−1^ l-alanine (Sigma), 0.2 g l^−1^ l-arginine (Sigma), 0.5 g l^−1^ l-asparagine (Sigma), 5 g l^−1^ soluble starch (Sigma), 2.5 g l^−1^ NaCl, 10 g l^−1^ NaSO_4_ and 20 g l^-1^ bacteriological agar (VWR)].

MAM agar was used for spore preparation [10 g l^−1^ wheat starch (Sigma), 2.5 g l^−1^ corn steep solids (Sigma), 3 g l^−1^ yeast extract (Fisher Scientific), 3 g l^−1^ CaCO_3_, 0.3 g l^−1^ FeSO_4_ 7H_2_O and 20 g l^−1^ bacteriological agar (VWR)].

Fermentation used SV2 [15 g l^−1^ glycerol, 15 g l^−1^ glucose, 15 g l^−1^ soy peptone (Sigma), 3 g l^−1^ NaCl and 1 g CaCO_3_] or EVL followed by EFL [EVL: 15 g l^−1^ corn steep solids, 30 g l^−1^ sucrose, 4 g l^−1^ (NH_4_)_2_ SO_4_ and 6 g l^−1^ CaCO_3_; EFL: 36 g l^−1^ Difco Select Soytone, 36 g l^−1^ corn starch, 2.4 g l^−1^ (NH_4_)_2_ SO_4_, 7.2 g l^−1^ CaCO_3_, 5 g l^−1^ soybean oil, 0.8 % w/v glucose and 0.0325% v/v propan-1-ol].

AVMM agar was used to interrogate utilization of specific nutrients: 1 g l^−1^ K_2_HPO_4_, 1 g l^−1^ KH_2_PO_4_, 0.1 g l^−1^ MgSO_4_, 10 mg l^−1^ FeSO_4_, 5 g l^−1^ asparagine, 20 g l^−1^ bacteriological agar (VWR) and 1% glucose. Variant AVMM omitted glucose and/or asparagine and replaced them with alternative sources of carbon and nitrogen.

Spore stocks were prepared by an established method [24]. Briefly, 200 µl of dense liquid culture (or 100–200 µl of an old spore suspension) was spread onto MAM agar plates, and recovered spores were resuspended in 15% glycerol and stored at −70 °C.

### Construction of engineered *S. erythraea*

GarA-H_6_ was cloned in shuttle vector pAW401, which integrates by *attP/B* [25], and has the strong constitutive *ermE* promoter (from the erythromycin resistance gene *ermE* of *S. erythraea*) [26] next to a multiple cloning site [27,28]. The *garA* gene, SACE_3845, 459 bp, was amplified from *S. erythraea* NRRL2338 genomic DNA using primers SKP1 (5′-AAAACATATAGCCACAACAGCGGCTCCG-3′) and SKP3 (AAAATCTAGAGCGGCCTTGTCCCCCGTCGAT) to add a C-terminal hexahistidine tag (H_6_-tag). GarA-H_6_ was inserted into the NdeI XbaI restriction sites of pAW401, and the plasmid was verified by sequencing with pAW401-specific primers (CGATGCTGTTGTGGGCACAATC,

AGTCCAAGCTCAGCTAATTAAGC). pAW401 carrying variant phosphoablative GarA-PB was generated by DpnI-mediated site-directed mutagenesis [29] with mutagenic primers (CGGAGCAGTCACCGGAGGCCGCCTCGGTCTTCAGGC, GCCTGAAGACCGAGGCGGCCTCCGGTGACTGCTCCG). The resulting constructs were introduced to *S. erythraea* by conjugation [30] with apramycin selection (50 µg ml^−1^) to produce transconjugants with integrated pAW401 (control or ‘Ctrl’), pAW401-GarA-H_6_ and pAW401-PB-GarA.

### Immunoblot detection of GarA

**Fig. 2.**
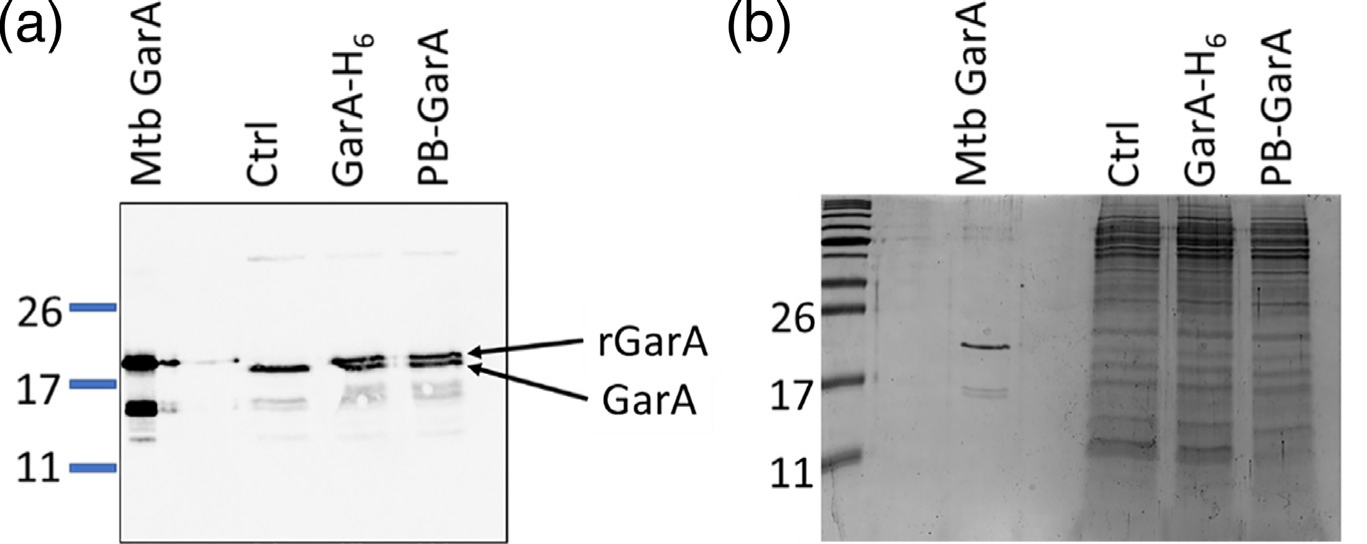
Engineered *S. erythraea* expressed GarA-H_6_ or PB-GarA at similar levels to endogenous GarA. (a) Transconjugant strains were lysed and analysed by SDS-PAGE and immunoblot with anti-GarA antibody. Arrows show recombinant GarA, ‘rGarA’, and endogenous GarA, ‘GarA’. (b) Protein loading for the immunoblot was normalized by Coomassie staining. To verify antibody specificity, a control strain without recombinant GarA was included (Ctrl) and pure recombinant *M. tuberculosis* H_6_-GarA (Mtb GarA).

**Fig. 3.**
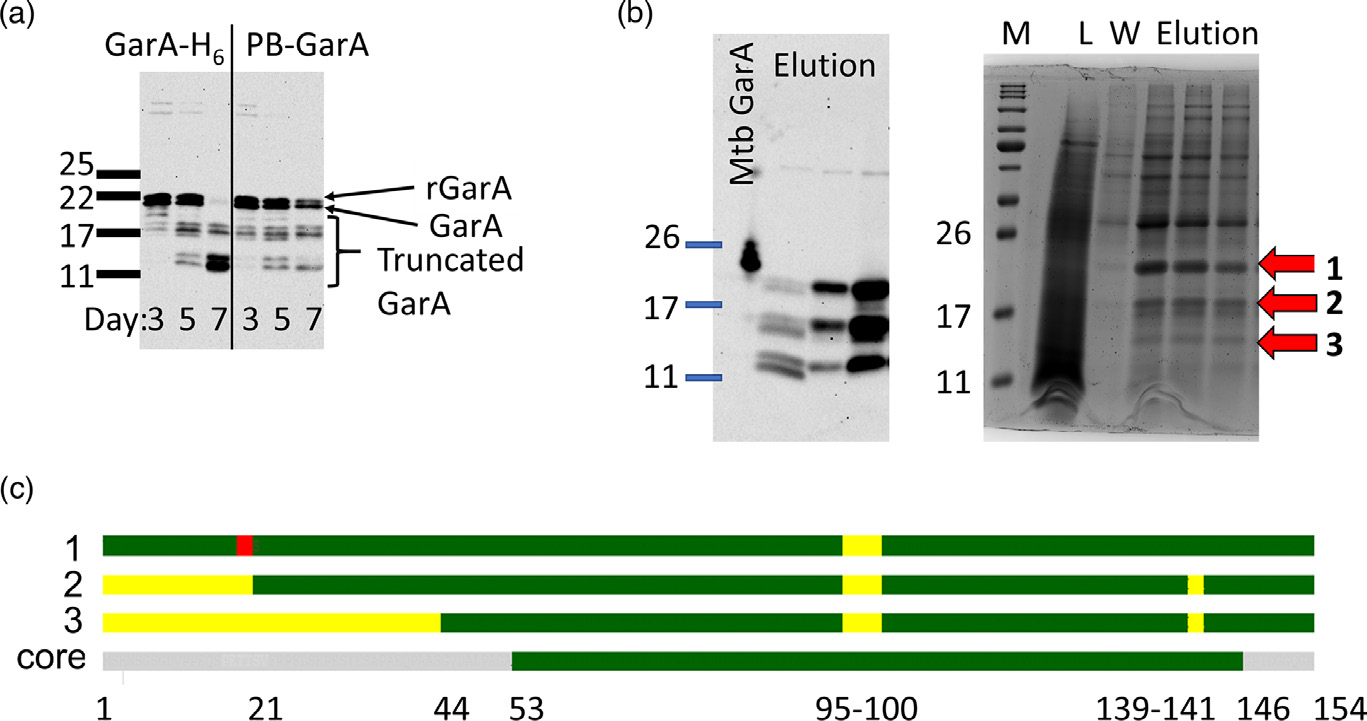
GarA lost its regulatory phosphorylation sites by truncation during fermentation. (a) *S. erythraea* transconjugants were cultured for the indicated time; then, lysates were probed by immunoblot using anti-GarA antibody, revealing full-length GarA and putative truncated GarA. (b) Lysate prepared as in panel (a) was subjected to immobilised metal affinity chromatography enrichment and analysed by immunoblot (left) and Coomassie-stained SDS-PAGE (right). L, lysate; W, wash; M, markers. Bands putatively identified as GarA are marked with arrows 1, 2 and 3. (c) Bands 1–3 were analysed by MS peptide fingerprinting. Green indicates peptides detected above the threshold (99%), and yellow indicates peptides not detected. For comparison, the core FHA domain is depicted in green, phosphorylation motif in red and residue numbers indicated underneath.

*S. erythraea* was cultured in SV2 medium for 3 days (Fig. 2) or 3–7 days (Fig. 3) and then harvested and lysed in PBS using bead beating in a FastPrep for 80 s. Following SDS-PAGE, an immunoblot with anti-GarA antibody was carried out as described [10]. Pure *M. tuberculosis* H_6_-GarA used as a positive control was made as described [7].

### Identification of truncated versions of GarA

Standard fermentation was carried out in SV2 medium for 7 days (five flasks of 50 ml), and bacteria were harvested by centrifugation and resuspended in 20 ml PBS with protease inhibitors. Cells were lysed by bead beating in a FastPrep for 80 s. After centrifugation, Gar-H_6_ was enriched from lysate using Ni-NTA column, and eluted fractions were analysed by SDS-PAGE gel with Coomassie staining and MS fingerprinting of tryptic peptides.

### Fermentation for erythromycin production

Fermentation followed a published method [24]. A seed culture was prepared by inoculating 7 ml of SV2 or EVL media with 70 µl of spore stock (normalized to 10^8^ spores/ml). For each engineered strain, seven individual transconjugants were analysed. Seed cultures were grown for 3 days at 28 °C shaken at 250 r.p.m.

For fermentation in SV2, 1.25 ml of seed culture was used to inoculate 25 ml of fresh SV2 medium in 125 ml conical flasks. For fermentation in EVL/EFL, 0.35 ml EVL seed culture was used to inoculate 7 ml of EFL medium in 50 ml screw capped tubes. Fermentations were incubated at 28 °C for 7 days with shaking.

### Erythromycin extraction

At the end of fermentation (day 7), 1 ml of bacterial culture was added to 1 ml of HPLC grade methanol and shaken on a Vibrax orbital shaker for 1 h. Samples were then centrifuged at 15,000 g for 20 min, and the supernatant was used for analysis.

### Determination of erythromycin titre by LC-MS

LC-MS was used to measure erythromycin concentration in extracted samples using a calibration curve prepared with commercially available erythromycin [31]. The column used was a reverse phase Xselect CSH C_18_, size 2.1 mm × 50 mm, particle size 3.5 µm (Waters) with an appropriate column guard. Flow was 1 ml min^−1^ at 60 °C using 95% water, 5% acetonitrile and 0.1% ammonium hydroxide solution (solvent A) and 95% acetonitrile, 5% water and 0.1% ammonium hydroxide (solvent B).

### Determination of erythromycin titre by bioassay

Fermentation culture was filter sterilized and then measured for erythromycin concentration by the bioassay disc diffusion method [32]. Bioassay used *Micrococcus luteus* (ATCC9341) exponential phase culture mixed in tryptic soy 0.4% top agar on tryptic soy agar plates. Filtered culture (10 µl) was soaked into sterile paper discs which were placed on the inoculated agar and incubated for 30 h at 30 °C in a static incubator before measuring zones of inhibition. Discs containing known amounts of pure erythromycin (Sigma) were used in parallel.

### Phenotypic characterization of engineered *S. erythraea* by growth on agar

AVMM has previously been used to study the utilization of different carbon and nitrogen sources by *Streptomyces* [4,24]. Standard AVMM contains a single nitrogen source, asparagine (38 mM) plus glucose (1%) as an additional source of carbon. Here, AVMM agar was prepared lacking asparagine and glucose, so that carbon and nitrogen sources could be tested individually. ‘Limited N’ contained 1% glucose but lacked an added nitrogen source. ‘Limited C’ contained 10 mM (NH_4_)_2_SO_4_ but lacked an added carbon source. ‘Glu as C’ contained 100 mM glutamate and 10 mM (NH_4_)_2_SO_4_. ‘Glu as N’ contained 1% glucose and 60 mM glutamine. ‘Gln as N’ contained 1% glucose plus 60 mM glutamine.

### RNA purification and quantitative PCR

RNA was extracted from 1 ml fermentation culture in SV2 medium at day 3 or day 7 by the addition of 2 ml RNAprotect™ (Qiagen) and harvesting by centrifugation. RNA was extracted using the RNeasy kit (Qiagen), using FastPrep bead beating for lysis, and DNA was removed using DNaseI. cDNA was generated using SuperScript III reverse transcriptase with random primer (NNNNNN) and RNaseOUT RNase inhibitor. qPCR used SensiFAST SYBR No-ROX kit (Qiagen). Controls were run for each set of primers: ‘no template’, ‘no reverse transcriptase’ and serial dilutions of *S. erythraea* genomic DNA. Primer pairs were designed to amplify regions from SACE_0721 (polyketide synthase, erythromycin cluster) TCGAGCTCTCCTGGGAAGT and GTCATCAGGTAGCCCTCGAC; SACE_0717 (sugar biosynthesis, erythromycin cluster) TCGACGACACCGCAGGGAGA and GTCAGCCAGTCGTGGGTTTCC; SACE_1801 (*sigA* housekeeping gene) TCTTGGCCGCAGAACTCTTG and TCCAGCTCCGCTGCAAACTC; and SACE_3845 (*garA*) ACAGCGACATCTTCCTGGAC and AACTTCCCGATCTGCACCT.

### Derivatization of aa from bacterial culture

Derivatization of aa standards used FDAA (1-fluoro-2-4-dinitrophenyl-5-l-alanine amide), also called Marfey’s reagent (Thermo Scientific) [33,34]. Fermentation was carried out in EFL medium, using three transconjugants for each strain, each grown in two flasks for 7 days. One millilitre of each culture was harvested by centrifugation at 15,000 g for 20 min. Pelleted bacteria were resuspended in 1 ml 1 : 1 HPLC grade MeOH/sterile water and shaken on a Vibrax orbital shaker for 1 h and then centrifuged. One hundred microlitres of supernatant were derivatized by the addition of 200 µl 1% FDAA in acetone followed by 40 µl of 1 M sodium bicarbonate with heating at 40 °C for 1 h with shaking. Twenty microlitres of 2 M HCl were added to each tube. After degassing, samples were diluted 1 : 2 with MeOH+0.1% HPLC grade formic acid. In parallel, aa standards were derivatized (100 µl of 5 µM aa; 11 most abundant proteinogenic aa, plus ornithine).

Derivatized aa were analysed by HPLC and detected by A_340_. Samples (5 µl) were injected through an Agilent 1100 HPLC equipped with a quaternary pump, using a reverse phase column – Gemini-NX C18 3µ 110A, size 150 mm × 4.6 mm, particle size 3 µm (Phenomenex). The flow rate was maintained at 1 ml min^−1^, and the temperature was set at 25 °C. Solvent A was 95% water, 5% acetonitrile and 0.1% formic acid, and solvent B was 95% acetonitrile, 5% water and 0.1% formic acid.

## Results

### Engineering of GarA expression in *S. erythraea*

To increase the expression of *garA* in *S. erythraea*, a hexahistidine-tagged version *garA*-H_6_ was cloned into an integrating vector for stable expression from a strong constitutive promoter. The tag was included to distinguish recombinant from native GarA by size and to facilitate detection and purification. In parallel, the region of *garA*-H_6_ encoding the phosphorylation sites was mutated to replace the threonines with alanine. This phosphoablative version, PB-*garA*, would avoid regulation by protein kinases. Transconjugants were obtained for *S. erythraea* carrying garA-H_6_, PB-garA and unmodified vector (‘control’). Immunoblotting detected the expression of endogenous GarA in all transconjugants, plus an additional band of higher molecular weight recombinant GarA in the transconjugants carrying *garA*-H_6_ and PB-*garA* but not ‘control’ (Fig. 2). The recombinant GarA caused only a modest increase in total cellular GarA (~2-fold), perhaps because GarA is naturally abundant. GarA has previously been found in the top 10% abundance for mRNA and proteins in transcriptomic and proteomic studies of *S. erythraea* and other Actinobacteria [35,36].

### GarA became truncated during the stationary phase, losing its regulatory kinase target site

While testing GarA expression by immunoblot, it emerged that older cultures of *S. erythraea* contained lower molecular weight bands (Fig. 3). GarA has previously been observed in a truncated form in *M. tuberculosis* [37], where the truncated form contains the entire FHA domain, making it competent for enzyme regulation but independent from control by kinases [12]. To confirm the identity of the putative truncated GarA-H_6_, it was enriched from cell extracts using metal affinity chromatography, separated by SDS-PAGE and analysed by MS (Fig. 3). The higher molecular weight band was confidently assigned as full-length GarA-H_6_ (97% coverage including both termini M1-R154) and the lower bands as N-terminal truncations lacking 20 or 42 residues but containing the entire FHA domain (Fig. 3).

Additionally, MS detected phosphorylation in the phosphorylation motif (ETTS) of full-length GarA (supporting information), confirming that *S. erythraea* GarA is targeted by protein kinases, similar to its homologues in *Mycobacterium* and *C. glutamicum*.

In summary, endogenous GarA expression was observed at all growth stages of *S. erythraea* and was regulated by phosphorylation during exponential growth but accumulated in a truncated, kinase-independent form in the stationary phase.

### Phosphoablative GarA increased erythromycin production

**Fig. 4.**
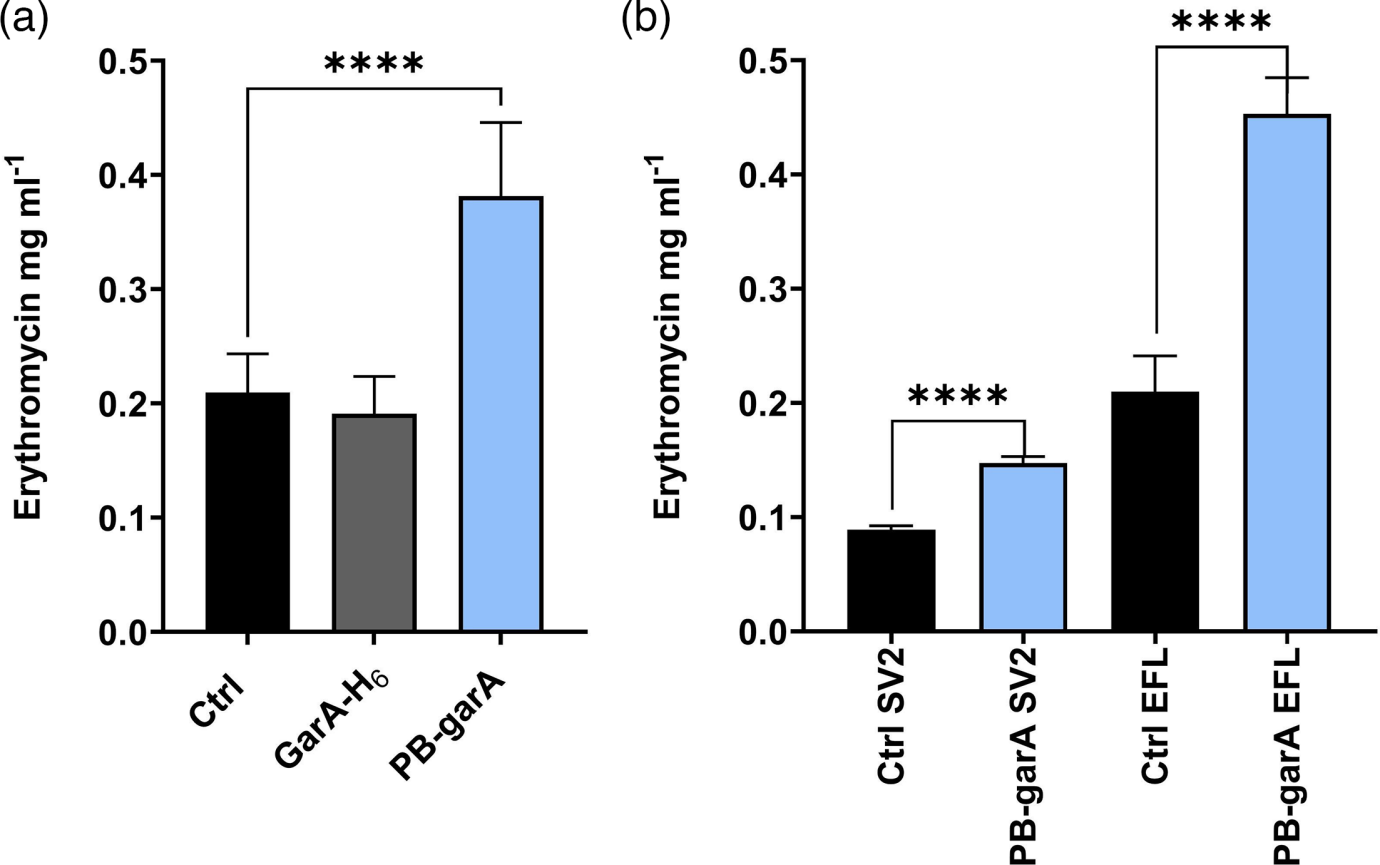
Erythromycin titre of *S. erythraea* expressing phosphoablative GarA was higher than that of the control strain. (**a**) Fermentation was carried out for 7 days in EFL broth; then, erythromycin was extracted and measured by LC-MS (mean and sd of erythromycin per millilitre of fermentation culture for seven transconjugants for each strain, each in triplicate flasks). The erythromycin titre from transconjugants expressing PB-GarA was significantly higher than that of the control strain (t-test, *P*<0.05), whereas there was no significant difference between the control and the strain expressing GarA-H_6_. (**b**) Fermentation was carried out for 7 days in EFL broth or SV2 broth, and filtered supernatant was analysed for erythromycin by bioassay (mean and sd for seven transconjugants of each). In both media, the erythromycin titre from PB-GarA was significantly higher than that of the control (t-test, *P*<0.05).

To evaluate the potential of GarA to increase erythromycin production, the engineered strains were cultured by batch fermentation and erythromycin was measured by liquid chromatography coupled with MS (LC-MS). The strain expressing phosphoablative GarA produced significantly more erythromycin than the control (twofold higher, *P*<0.05), whereas the strain expressing GarA-H_6_ was not significantly different from the control (Fig. 4a).

To validate the engineered increase in erythromycin titre, fermentations were repeated and assessed by an independent method (bioactivity assay) and with an alternative fermentation broth (Fig. 4b). In all cases, the PB-GarA strain produced significantly more erythromycin than the control (Fig. 4).

### Phosphoablative GarA affected nutrient utilization

**Fig. 5.**
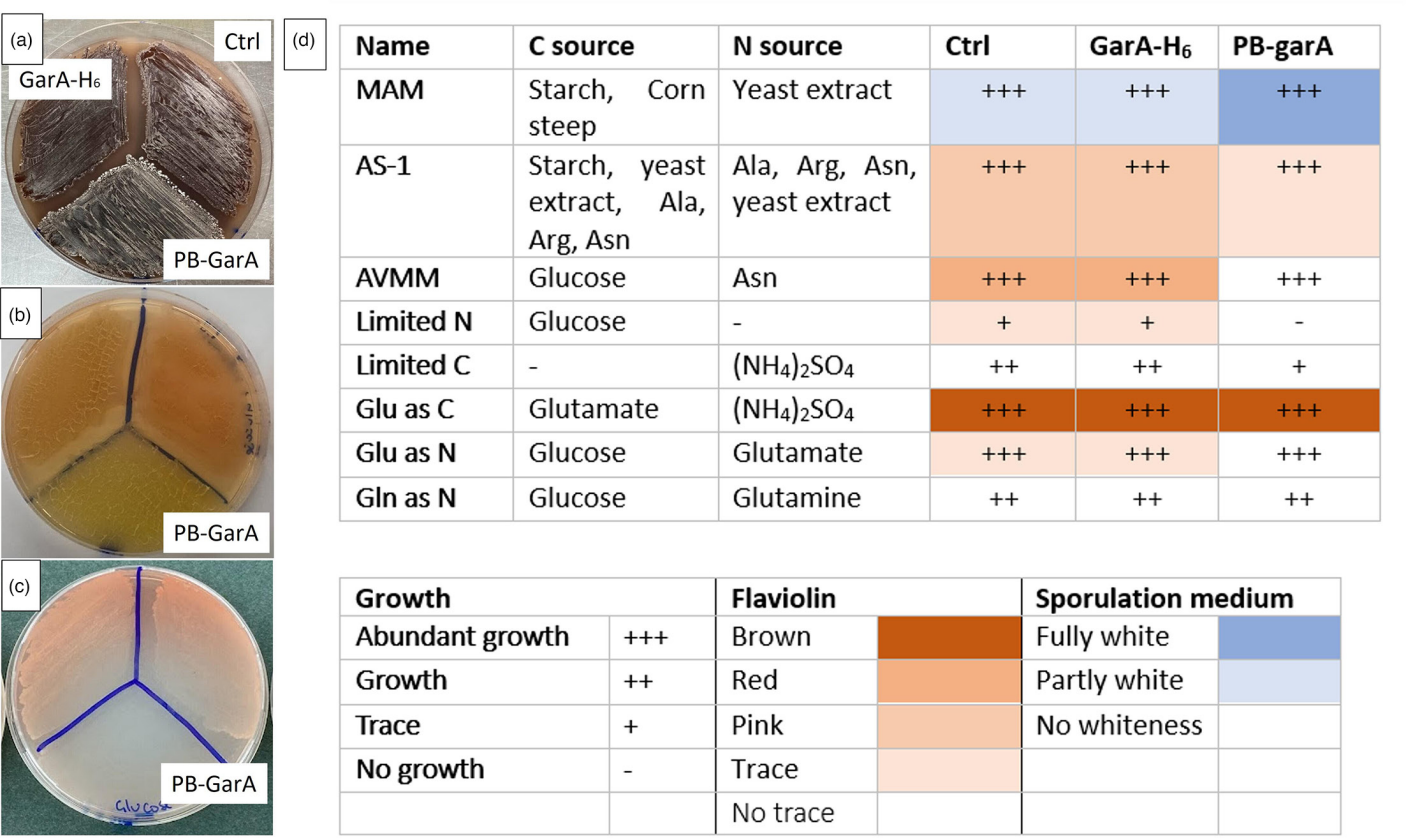
PB-GarA led to nutrient-specific changes in flaviolin production and growth. ‘Ctrl’ indicates the control strain with an unmodified vector. (**a–c**) *S. erythraea* transconjugants of each strain were streaked on agar-based medium. Photos are representative of three experiments each using independent transconjugants. (**a**) PB-GarA strain showed earlier appearance of white colouration, characteristic of sporulation, on MAM plates. (**b**) PB-GarA strain showed less production of red/brown flaviolin on AVMM plates with glucose/asparagine. (**c**) PB-GarA strain was unable to grow on AVMM plates that lacked nitrogen supplementation. (**d**) Results of all plates were summarized in table form using qualitative scoring for amount of growth, pigment and whiteness explained in the key. MAM was used as a sporulation medium.

Apart from the erythromycin yield, the effects of engineered GarA on phenotype were investigated by comparing the engineered strains for their growth and morphology on a variety of agar-based media. The strain expressing GarA-H_6_ resembled the control in all conditions tested, whereas the strain expressing PB-GarA differed from the control on several growth media: reduced growth under nutrient limitation, reduced pigmentation in a subset of media and possible early sporulation (Fig. 5). Minimal media recipes were guided by the metabolic defects of *M. smegmatis* carrying phosphoablative GarA, which fails to catabolize glutamate efficiently [10]. Unlike mutant *M. smegmatis*, PB-GarA *S. erythraea* was able to catabolize glutamate, perhaps due to its larger genome containing additional paralogues of glutamate synthase and glutamate dehydrogenase [38]. The red-brown pigmentation is due to the polyketide flaviolin, which is synthesized from malonyl-CoA [39]. Levels of flaviolin varied strongly with nutrient source and may reflect levels of intracellular malonyl-CoA [40].

### Effects of PB-GarA on the expression of the erythromycin biosynthetic gene cluster

**Fig. 6.**
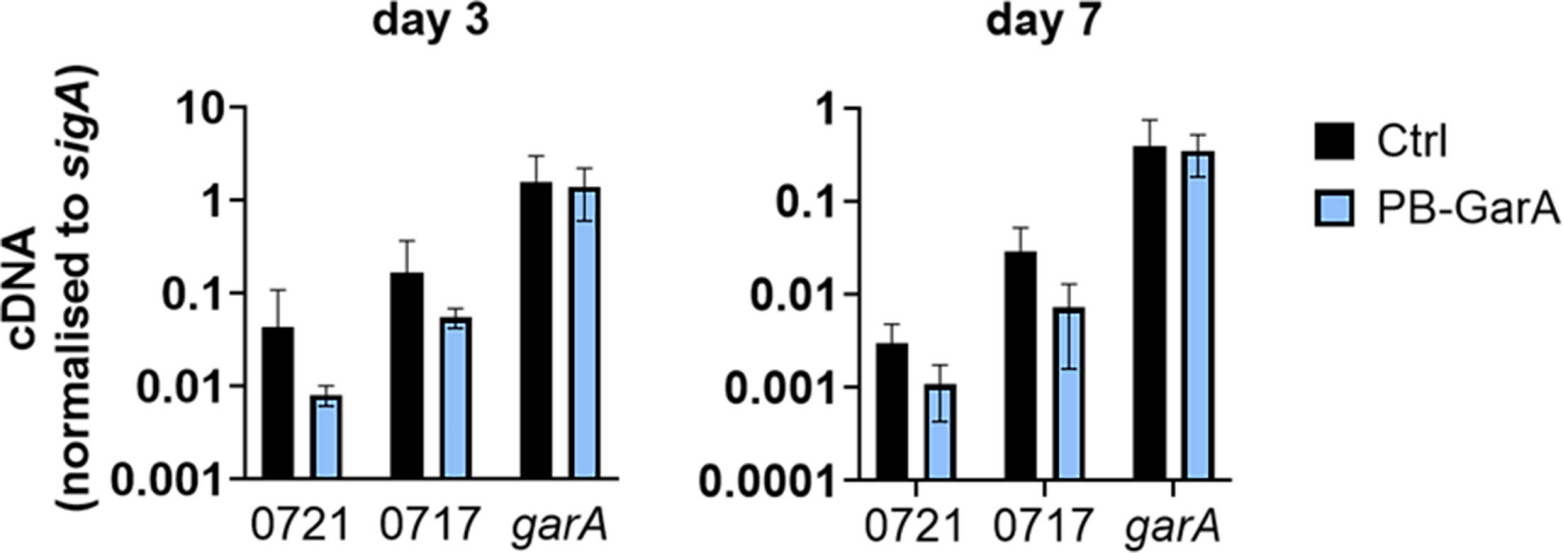
PB-GarA did not have a significant effect on the transcription of the erythromycin biosynthetic cluster. cDNA was prepared from three transconjugants of each strain after 3 or 7 days of fermentation in SV2 medium for reverse transcription and quantitative PCR. Graphs show the mean and sd cDNA copy number normalized to *sigA*. Gene expression in the PB-GarA strain did not differ significantly from the control strain carrying an unmodified vector (Ctrl) for any gene at either time point (t-test, *P*>0.05). SACE_721 and SACE_0717 are genes in the erythromycin biosynthesis cluster.

The predicted effects of PB-GarA on aa metabolism and the TCA cycle could influence the erythromycin biosynthesis by direct effects on acyl-CoA supply for biosynthesis, or indirect effects on the expression of the biosynthetic gene cluster since nutritional limitation is known to regulate differentiation and natural production expression [41]. To determine whether PB-GarA caused an increase in transcription of the cluster, primers were designed for quantitative PCR of the polyketide synthase gene SACE_0721 and a glycosyl transferase gene SACE_0717 from the cluster, as well as *garA* itself. There was no significant difference between the PB-GarA strain and the control strain for any of these genes at day 3 or 7 of fermentation (Fig. 6).

### PB-GarA caused elevated levels of intracellular aa

**Fig. 7.**
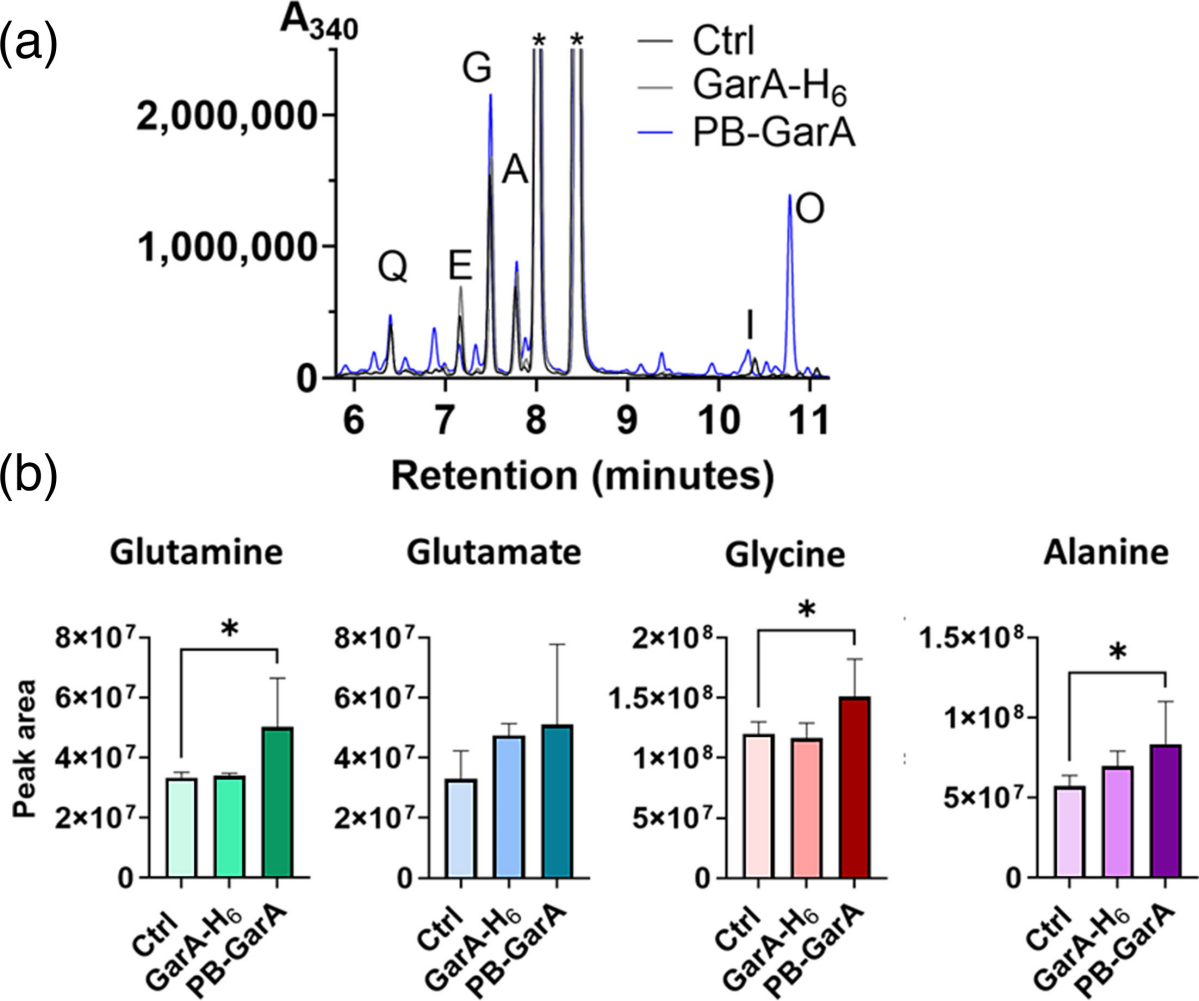
PB-GarA altered intracellular aa concentrations of *S. erythraea*. Intracellular metabolites were isolated from three transconjugants for each strain (two flasks per transconjugant in EFL medium for 7 days) and treated with Marfey’s reagent to derivatize the aa, followed by HPLC. Panel (a) shows example traces for one flask for each strain. Peaks corresponding to glutamine, glutamate, glycine, alanine, isoleucine and ornithine were identified by comparison to standards. Other, less abundant aa are not labelled on the trace. Residual unreacted Marfey’s reagent gave two peaks, labelled with an asterisk (*). Panel (b) compares the aa concentration in extracts from each strain, calculated from the area under the HPLC curve (mean and sd of six flasks, Ctrl compared with PB-GarA by t-test; * indicates *P*<0.05).

To better understand how PB-GarA affects the metabolism of *S. erythraea* in fermentation conditions, samples from fermentation flasks were analysed for intracellular aa by derivatization and HPLC. Glycine, alanine, glutamate and glutamine were the most abundant aa in all strains and were at elevated concentration in PB-GarA cells compared with control (*P*<0.05 for glutamine, glycine and alanine, Fig. 7). PB-GarA also contained additional peaks compared with other strains, including a putative peak for ornithine present in three out of six biological replicates, and there was greater flask to flask variation for PB-GarA than control of GarA-H_6_ strains (seen as larger sd in Fig. 7). Peaks were identified by comparison of retention time with standards, and ornithine was included as a standard as it was one of the most upregulated metabolites in *M. smegmatis* expressing PB-GarA [10]. Overall, HPLC analysis showed that PB-GarA caused significant changes in aa metabolism and increased abundance of aa that are consistent with the effects of engineering GarA in other Actinobacteria [6,10].

## Discussion

Engineered *S. erythraea* expressing phosphoablative GarA was enhanced for erythromycin production and displayed metabolic changes consistent with GarA function as a TCA cycle regulator. The twofold increase in erythromycin yield in an overproducer strain was significant and was observed in two growth media and using two independent methods to measure antibiotic production. This improvement is comparable with other genetic methods applied to *S. erythraea*, which typically enhance yields by 50–70% [32,42, 43]. Overall, the observed titre of 200 mg l^−1^ was comparable to other studies using flask fermentation [2,32], though lower than bioreactor [42].

The onset of antibiotic production can be co-regulated with the developmental changes of sporulation [44]. Consistent with this, the engineered strain may potentially undergo early sporulation. PB-GarA could potentially affect sporulation by increasing intracellular alpha-ketoglutarate (since it inhibits ODH). Various bacteria sense the ratio of alpha-ketoglutarate to glutamine as a measure of nitrogen availability [45], and nitrogen limitation can stimulate both sporulation and antibiotic production [46]. Impaired sensing or regulation might also explain the observed growth impairment of PB-GarA strain during nutrient limitation. Interestingly, GarA was found to be predominantly truncated at late stages of fermentation, meaning that it would be independent from regulation by kinases. This mirrors observations in *Mycobacterium* and *C. glutamicum* where phosphorylated GarA predominates during growth, and unphosphorylated GarA predominates in minimal media, starvation and stationary phase to repress the TCA cycle [10,11], and is consistent with metabolic switching when *Streptomyces coelicolor* experiences nutrient depletion [47].

*S. erythraea* encodes more than one polyketide biosynthase gene cluster, including the flaviolin cluster for synthesis of the red-brown pigment. These clusters potentially place competing demands on the cellular acyl-CoA pool. Erythromycin is synthesized from methylmalonyl-CoA and propionyl-CoA, while flaviolin is synthesized from malonyl-CoA [39]. The acyl-CoA supply is an important determinant of polyketide yields: deletion of unwanted polyketide synthases can increase the yield of desired polyketides [30], and increasing the expression of acyl-CoA carboxylases can also increase polyketide production [48]. The reduction in flaviolin production by the engineered PB-GarA strain likely indicates changes in acyl-CoA metabolism, including reduced intracellular malonyl-CoA [40], which is a potential mechanism for the increase in erythromycin yield.

**Fig. 8.**
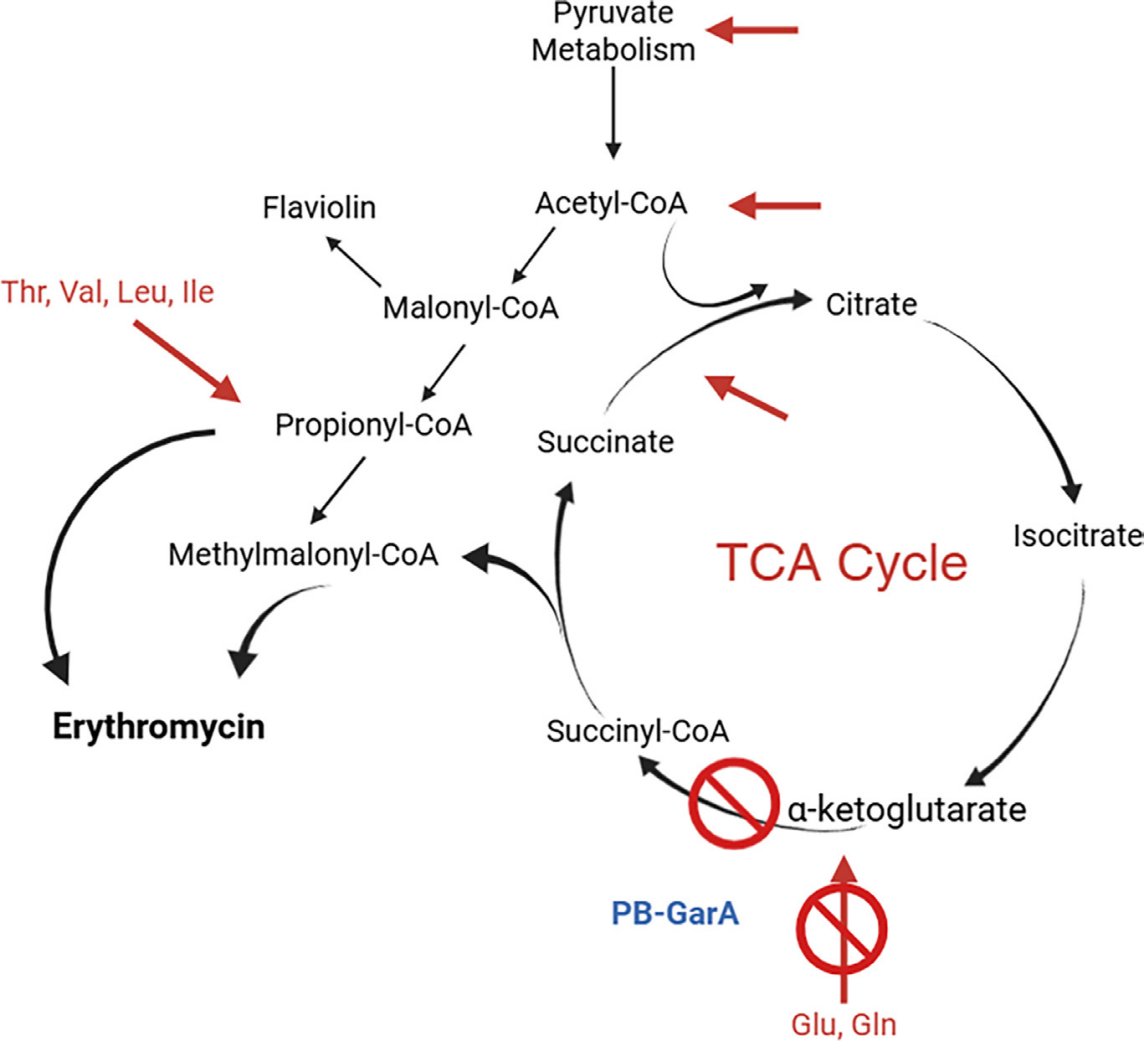
PB-GarA increased intracellular aa, which could enhance the availability of acyl-CoA precursors for erythromycin biosynthesis. PB-GarA inhibits the catabolism of glutamate through the TCA, which increased the concentration of many intracellular aa. Pathways for aa catabolism are shown with red arrows and can proceed via acyl-CoAs. Notably, Thr, Val, Leu and Ile can produce propionyl-CoA and methylmalonyl-CoA, the precursors for erythromycin biosynthesis.

Other changes in metabolism were apparent from HPLC: intracellular aa were generally higher in the engineered strain, similar to metabolome alterations in GarA-PB-engineered *M. smegmatis* [10]. This is consistent with the known functions of GarA that increase intracellular glutamate by inhibiting glutamate catabolism via the TCA cycle and activating glutamate synthase [12]. Since glutamate is the main donor/product in transamination reactions in the synthesis/breakdown of other aa, changes in glutamate metabolism would have wide-ranging effects on intracellular aa pools and, hence, on acyl-CoAs that are produced during aa catabolism (Fig. 8).

Only the strain with phosphoablative GarA exhibited changes in phenotype, while the strain with GarA-H_6_ matched the control. We attribute this to the action of protein kinases, which could offset the extra GarA by inactivating it by phosphorylation. Interestingly, kinase regulation of GarA appeared to be less important or absent during late fermentation, when a truncated, constitutively active form of GarA predominated. It is tempting therefore to speculate that the increase in erythromycin yield in the PB-GarA strain could be due to changes in metabolism early in fermentation, for example, an earlier metabolic switch.

This study offers an insight into the role of GarA in regulating primary metabolism and the production of secondary metabolites in *S. erythraea*, contributing to a broader understanding of metabolic regulation in Actinobacteria. Additionally, the results and methodologies presented here suggest a promising strategy for improving the yield of other bioactive molecules by engineering GarA to favour the desired biosynthesis.

## Supplementary material

10.1099/mic.0.001583 Supplementary Material 1.

## Abbreviations

FHA: forkhead associated
LC-MS: liquid chromatography coupled with MS
ODH: oxoglutarate dehydrogenase
