# Supplementary material for: Engineering the TCA cycle regulator GarA to increase erythromycin production in Saccharopolyspora erythraea
Source: Microbiology (Reading). 2025 Aug 4;171(8):001583. doi: 10.1099/mic.0.001583 (PMC12321487; doi:10.1099/mic.0.001583)
Supplement: Supplementary Material 1. [file mic-171-01583-s001.pdf]

Supporting information - Engineering the TCA cycle regulator GarA to increase erythromycin production in *Saccharopolyspora erythraea*

Mass spectrum LC MSMS and fragment table of GarA-H_6 showing phosphorylation in the sequence ETTS.

The table shows all relevant B ions and Y ions. The predicted ions that were detected within 0.020 AMU are shaded in colour in the table (red for B ions, green for B ions that lost water, blue for Y ions).

The associated spectrum is coloured to match the table. The detected y10 and y15 ions (fragment sequences PE**TT**SVFRPF and PPEQSPE**TT**SVFRPF) indicate that the peptide is a phosphopeptide (80 AMU higher than the weight predicted if fragments were not phosphorylated) and the detected ions y2, y3, y4, y5 and y6 (fragment sequence of y6 = SVFRPF) indicate that the location of the phosphorylation is one of the threonines in the region ETTS (since none of the ions y2-y6 have the 80 additional AMU attributed to phosphorylation).

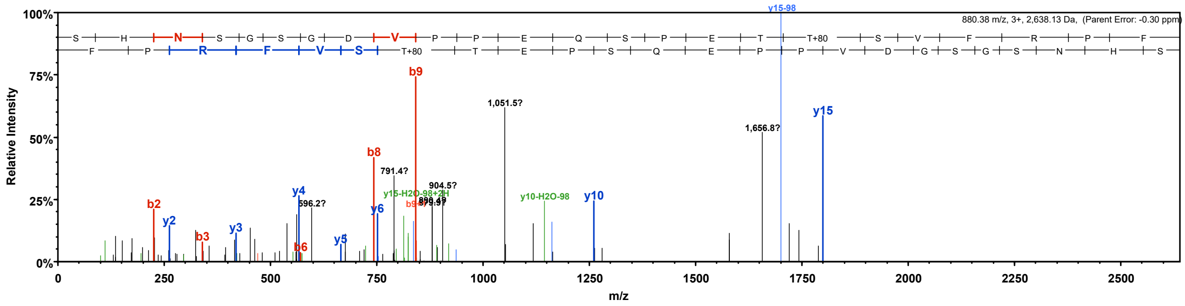

B	B Ions	B+2H	B-NH3	B-H2O	AA	Y Ions	Y+2H	Y-NH3	Y-H2O	Y
1	88.0	44.5		70.0	S	2,639.1	1,320.1	2,622.1	2,621.1	24
2	225.1	113.1		207.1	H	2,552.1	1,276.6	2,535.1	2,534.1	23
3	339.1	170.1	322.1	321.1	N	2,415.1	1,208.0	2,398.0	2,397.0	22
4	426.2	213.6	409.1	408.2	S	2,301.0	1,151.0	2,284.0	2,283.0	21
5	483.2	242.1	466.2	465.2	G	2,214.0	1,107.5	2,196.9	2,196.0	20
6	570.2	285.6	553.2	552.2	S	2,157.0	1,079.0	2,139.9	2,138.9	19
7	627.2	314.1	610.2	609.2	G	2,069.9	1,035.5	2,052.9	2,051.9	18
8	742.3	371.6	725.2	724.3	D	2,012.9	1,007.0	1,995.9	1,994.9	17
9	841.3	421.2	824.3	823.3	V	1,897.9	949.4	1,880.8	1,879.9	16
10	938.4	469.7	921.4	920.4	P	1,798.8	899.9	1,781.8	1,780.8	15
11	1,035.4	518.2	1,018.4	1,017.4	P	1,701.8	851.4	1,684.7	1,683.7	14
12	1,164.5	582.7	1,147.5	1,146.5	E	1,604.7	802.9	1,587.7	1,586.7	13
13	1,292.6	646.8	1,275.5	1,274.5	Q	1,475.7	738.3	1,458.6	1,457.6	12
14	1,379.6	690.3	1,362.6	1,361.6	S	1,347.6	674.3	1,330.6	1,329.6	11
15	1,476.6	738.8	1,459.6	1,458.6	P	1,260.6	630.8	1,243.5	1,242.6	10
16	1,605.7	803.3	1,588.7	1,587.7	E	1,163.5	582.3	1,146.5	1,145.5	9
17	1,706.7	853.9	1,689.7	1,688.7	T	1,034.5	517.7	1,017.4	1,016.5	8
18	1,887.7	944.4	1,870.7	1,869.7	T+80	933.4	467.2	916.4	915.4	7
19	1,974.8	987.9	1,957.7	1,956.8	S	752.4	376.7	735.4	734.4	6
20	2,073.8	1,037.4	2,056.8	2,055.8	V	665.4	333.2	648.4		5
21	2,220.9	1,111.0	2,203.9	2,202.9	F	566.3	283.7	549.3		4
22	2,377.0	1,189.0	2,360.0	2,359.0	R	419.2	210.1	402.2		3
23	2,474.1	1,237.5	2,457.0	2,456.1	P	263.1	132.1			2
24	2,639.1	1,320.1	2,622.1	2,621.1	F	166.1	83.5			1
